# Tools Used to Measure the Physical State of Women with Celiac Disease: A Review with a Systematic Approach

**DOI:** 10.3390/ijerph17020539

**Published:** 2020-01-15

**Authors:** Alejandro Martínez-Rodríguez, Daniela Alejandra Loaiza-Martínez, Javier Sánchez-Sánchez, Pablo J. Marcos-Pardo, Soledad Prats, Fernando Alacid, Jacobo A. Rubio-Arias

**Affiliations:** 1 Faculty of Sciences, University of Alicante, 03690 Alicante, Spain; maria.prats@ua.es; 2 Faculty of Sports, Catholic University of Murcia (UCAM), 30107 Murcia, Spain; daloaiza@alu.ucam.edu (D.A.L.-M.); pmarcos@ucam.edu (P.J.M.-P.); 3 School of Sport and Science, European University of Madrid, 28670 Madrid, Spain; javiersanchezsanchez22@gmail.com; 4 IGOID Research Group, University of Castilla de la Mancha, 13071 Castilla la Mancha, Spain; 5 Department of Education, Health Research Centre, University of Almería, 04120 Almería, Spain; falacid@ual.es; 6 LFE Research Group, Department of Health and Human Performance, Faculty of Physical Activity and Sport Sciences, Universidad Politécnica de Madrid (UPM), 28040 Madrid, Spain; jacobo.rubio2@gmail.com

**Keywords:** diagnosis, diet, gluten, symptoms, women

## Abstract

Celiac disease (CD) is an immunological disorder that mainly affects the small intestine, generating an inflammatory process in response to the presence of gluten (a protein). Autoimmune diseases are part of a group of diseases that are difficult to diagnose without a specific protocol or consensus to detect them due to the number of symptoms and diseases with which it has a relationship. Therefore, the aim of this review was to analyze the diagnostic tools of CD used in middle-aged women, to compare the use and effectiveness of the different tools, and to propose a strategy for the use of the tools based on the results found in the literature. The present research followed the Preferred Reporting Items for Systematic Reviews and Meta-Analyses (PRISMA) guideline. The search was conducted in the following databases: Scielo, PubMed, Web of Science, and Worldwide Science org. In the initial literature search, 2004 titles and relevant abstracts were found. Among them, 687 were duplicates, leaving 1130 articles. Based on the inclusion criteria, only 41 articles passed the selection process; 4 main types of analyses appear in the studies: blood tests, questionnaires, clinical history, and biopsy. It can be said that none of the analyses have a 100% reliability since most of them can present false negatives; therefore, the best way to diagnose celiac disease up to now is through a combination of different tests (Immunoglobulin A and small intestinal biopsy).

## 1. Introduction

Celiac disease (CD) is an immunological disorder that mainly affects the small intestine, generating an inflammatory process in response to the presence of gluten (a protein found mainly in wheat, barley, and rye, among other contaminated foods like oatmeal) [1,2,3,4]. It is currently estimated that 1% of the world population suffers from CD; it mainly occurs in women, with a probability of 2:1 [3,5]. The average age for diagnosis is around 45 years in Europe in the last 10 years; in Spain, the last analysis said that most of the diagnoses occur in persons over 20 years. However, the average does not mean that it cannot be diagnosed earlier; it means that, in this decade, more diagnoses happen around that age. Multiple factors can be related to the diagnosis age, such as no symptoms, ignorance, or even no presence of intestinal inflammation [6]. This disease is related to weight variation, hepatobiliary, and neurological or endocrine disorders, such as hypothyroidism [1]; it has also been linked to problems of infertility and affects the absorption of nutrients such as calcium and vitamin D (the main causes are the change in the small intestinal villi that can no longer absorb all vitamins and nutrients and the lower vitamin D levels related to low exposure to sunlight that a large part of the population experiences, whether due to changing seasons or to working in closed spaces for long hours); therefore, CD is associated with the reduction of bone mass [7,8,9,10].

The only effective treatment is a gluten-free (strict) diet. With the disappearance of the symptoms, the serology normalizes and the intestinal villi recover. The abandonment of treatment can cause complications that, especially in adulthood, can manifest as osteopenia, osteoporosis, and high risk of neoplasms in the digestive tract [11,12,13]. Also, the perception of the intensity of hot flushes and irritability is more serious in celiac (undiagnosed or poorly treated) women with menopause [14] because middle-aged women are the most affected by the consequences of not having a gluten-free diet amongst people with celiac disease [14]. The investigation focuses on the studies conducted in this population because, for different causes such as false negatives, a lot of celiac women are underdiagnosed.

Nutrition has an important role in these patients because a patient that fails to maintain a gluten-free diet does not absorb the necessary nutrients which can then cause problems like anemia or osteoporosis; this is because, when the patient consumes gluten, the nutrients are not absorbed by the body because the reaction to gluten ends with a flat intestinal villus [15]. Also, constant cell division could even result in malignant neoplasm caused by the renovation of intestinal cells more times than it should in a normal life [15].

At the same time, all the tools that a health personnel should use to know the status of patients in their entirety is not clear, since it involves not only the physical state but also the psychological state and the disease damages multiple organs. Autoimmune diseases are part of a group of diseases that are difficult to diagnose [16] because some of the symptoms are really common and the health personnel does not request very invasive diagnostic tests or tests with high risks such as biopsies without the patient having enough clear symptoms of the disease. In this disease, the clinical history of the patients is very important to clarify symptoms that could go unnoticed [15,17,18]. Therefore, a guide with different analyses can clarify the diagnosis and help avoid false negatives. Therefore, our aim is to analyze the diagnostic tools of CD used in middle-aged women, to compare the use and effectiveness of the different tools, and to propose a strategy for the use of the tools based on the results found in the literature.

## 2. Materials and Methods

### 2.1. Data Sources and Searches

The present research followed the Preferred Reporting Items for Systematic Reviews and Meta-Analyses (PRISMA) guideline. The search was conducted from January 2018 until 1 May 2018 in the following databases: Scielo, PubMed, Web of Science, and WorldWide Science org. The following MeSH (Medical Subject Headings, is the National Library of Medicine controlled vocabulary thesaurus used for indexing articles for PubMed) [19] search headers were used: women and celiac, coeliac, “gluten free”, or gluten. Subsequently, a second search was made on 25 February 2019. The eligibility criteria were predetermined by the authors. The search was registered on the Prospero data base (Registration number CRD42019125224).

### 2.2. Inclusion and Exclusion Criteria

The inclusion criteria are the following: studies performed in celiac patients, published in scientific journals, using tools to measure the health status of patients, which can be found as a full article, published less than 10 years ago, written in English or Spanish, and performed on human beings.

On the other hand, the exclusion criteria were the noninclusion of women, the study not containing any measuring tool in celiac patients, the non-identification of a complete article, and the age of the patients being less than 18 years. Retrieved articles were reviewed independently by two authors (D.A.L.-M and A.M.) to choose potentially relevant articles; all disagreements were solved by a third reviewer (J.A.R.A.). Two reviewers (D.A.L.-M and A.M.) independently extracted data from the included studies. The following information was extracted: site and country of the study, type of exercise, number of women included, age, tools or analysis used, and clinical characteristics. This review’s target was a useful framework for thinking about patient outcomes attributable to medical testing, and the focus was on outcomes that result from clinical management of CD based on the test results and on the direct health effects or diagnosis of CD testing.

The variables reviewed were all those analyzed, and tools used to determine the health status of a celiac woman and variables such as age, pathology, and gender of the patient were also reviewed. The extraction of data was performed independently by P.S. and S.A., and conflicts were resolved by consensus. The PEDro scale (an scale made to measure the quality of reports) was performed by the reviewers (D.A.L.-M and A.M.) to all the selected articles.

### 2.3. Analisis of the Risk of Bias

One of the biases considered in this study was that of working with women since various male symptoms are excluded from the study; in turn, as it is the group with the highest incidence and problems caused, it makes sense to give special importance to their symptoms. Another likely bias is the appearance of new methods and improved analyses in the last year, which could not be seen in this study or in our approach.

Another risk of bias is the population (country and age) and the amount of persons in the studies because, in the case of control, more tests could been made to compare with other studies but, in this case, we are not comparing the results, only the methods.

In turn, it should be noted that the studies used also presented a risk of bias, since in some cases, they had worked with the European population specifically or with cases treated in public hospitals exclusively but we do not consider that they are an important bias for the meta-analysis.

## 3. Results

### Characteristic of the Included Studies

**Figure 1:**
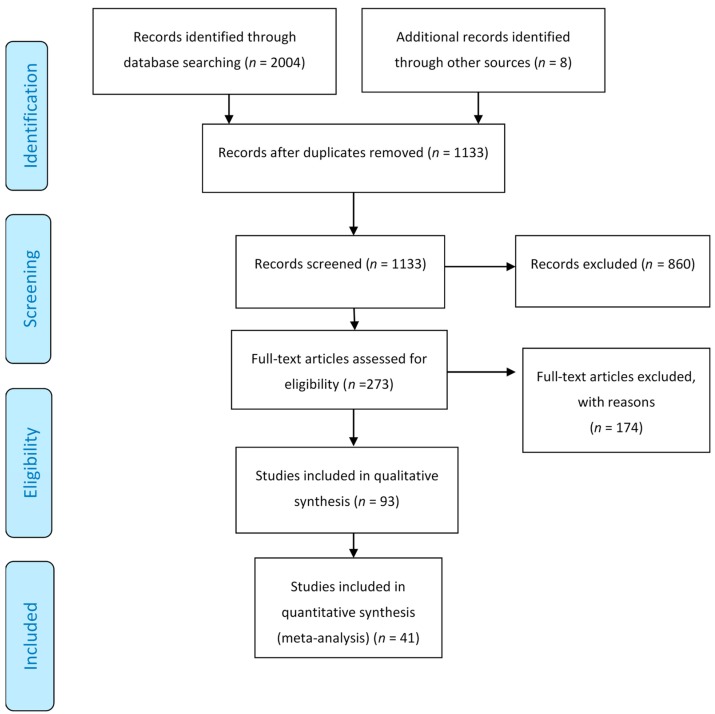
Flow diagram of the process of study selection.

In the initial literature search, 2004 titles and relevant abstracts were found. Among them, 687 were duplicates, leaving 1130 articles. A total of 860 were excluded based on abstracts and title screening. Full texts were retrieved from the remaining 270 articles. Based on the inclusion criteria, only 41 articles passed the selection process. Figure 1 shows the flow diagram of the study.

In Table 1, all the articles used for the review are presented, including the number of patients, the journal impact index, the age of the participants, and the kind of tests that the studies used.

In 24 of the studies found, they used intestinal biopsy as the main source of diagnosis of celiac disease, followed by blood tests of immunoglobulin A, anti-inflammatory transglutaminase, and Immunoglobulin G [4,5,9,10,13,19,22,24,27,28,30,31,32,39,40,41,43,45,46,49,50,51]. The relevant information was explained in the Table 2; Table 3.

Another of the least performed studies for the diagnosis of CD is the immunoassay metric [40]; in this, the level of antigens and antibodies is measured and they use the serum endomysia antibody [1,19,20,22,33,35,37]. Different articles present that sensitivity to gluten or development of CD were related to a genetic factor.

In the different studies, factors that help to measure fertility were measured, such as the anti-Müllerian hormone, since we speak of middle-aged adult women [39]. They also used routine physical examinations such as weight [5,7,10,20,26,27,32,51], height [5,7,10,20,26,27,32,51], body mass index (BMI, a formula that uses weight and height) [5,7,10,20,26,27,32,51], fat mass, and energy consumption [4,5], which were compared with their clinical analysis to be able to complement the patient’s profile and to make a diagnosis. Analysis of the food consumed can also be found in order to help the patient with his or her nutritional treatment, which is the only known treatment for CD [5].

Different tests that help the health personnel to know the physical and psychological states of the patients with CD have been found in the different studies and is very important because it affects the adherence to treatments for a good state of health [5,52,53].

In all the articles, the clinical history is an important document for diagnosis because it helps to determine or find different symptoms that could be unnoticed by the patients; energy consumption appears in a few studies to determine their nutrition, as do other data such as weight, body mass, body mass index, and fat mass [15,17,18,54,55].

## 4. Discussion

It can be seen that, of the 41 articles found, the main diagnostic method of CD is biopsy but not before subjecting the patient to blood tests that show an elevation of immunoglobulins [4,5,9,10,20,22,24,26,27,30,31,32,37,39,41,43,45,46,49,50,51,58]. In Italy, a study shows that the prevalence of spontaneous abortion in celiac women was higher than in non-celiac women [29]. Another study made in United States concluded that women with unexplained infertility have more risk of having CD [38]. A meta-analysis published in 2014 concluded that undiagnosed CD is a risk factor for infertility [8]. In a study made in the United Kingdom, it was concluded that celiac women have the same possibilities of the present fertility problems [7].

As the biopsy is an invasive analysis which entails risks, some alternatives are proposed for those patients who present symptoms: serological studies, biomarkers, and EMA (Endomysial antibodies) analysis [59,60]. The biggest problem with EMA analysis is that it must be accompanied by serological samples. On the other hand, biomarkers fail in 20% of cases; although they would be a great way to detect CD, they are not yet sufficiently reliable [59,60].

When making a diagnosis of CD, it is very important to know if the patient suffers from the different symptoms; since studies that diagnose CD are invasive analyses [4,5,9,10,20,22,24,26,27,30,31,32,37,39,41,43,45,46,49,50,51,58]. Therefore, before requesting the analyses, it is useful to perform a Celiac Symptom Index test to identify the symptoms apart from the clear use of the clinical history; in this way, a better diagnosis can be made [57].

Metabolomics is an analysis that rapidly evolves and allows a metabolic “fingerprint” of patients with celiac disease with low-invasive and low-risk tests, which in part have a low cost [56]. This study uses different techniques, among the least invasive to diagnose celiac disease, we can find the tests performed with feces, with the technique of PCR-DGGE (real-time polymerase chain reaction, denaturing gradient gel electrophoresis) [61].

When talking about avoiding different diseases caused by the lack of treatment of CD; it is important to consider the quality of life and the psychological state of the patients in addition to compliance with the diet, since factors such as anxiety, depression, and poor quality of life can cause patients to abandon their treatment or can interrupt them from time to time [5,52,53,58]. Despite this, the tools to measure the psychological state of patients are generally not used due to lack of knowledge or time while consulting. Therefore, studies in which they use this type of test try to promote its use and make known the benefits of it. For all this, it is important that they are among the different diagnostic tools in celiac disease.

To avoid false negatives in the diagnosis results for CD, the diet of the patient is important because the gluten-free diet shows an improvement in all the analyses and could pass as negative. Because of that, the patients to be analyzed should eat gluten the months before a biopsy or a blood test, the main problem is the risk (malignant neoplasm) and the health issues that this carries (diarrhea, malabsorption of nutrients, headaches, and anemia, for example).

During the search for articles, the reviewers (D.A.L.-M and A.M.) found a series of limitations, such as articles that did not appear because they did not contain the word celiac disease since they included it in immunological or intestinal diseases; the constant appearance of different tools for health status analysis that are simply the old ones with different names; and the constant publication of new articles that force us to be in constant education and to search for tools.

This study provides a guide to facilitate the diagnosis and to check the health status of middle-aged celiac patients, thereby reducing the time of search for diagnosis, the amount of analysis, and with this, the risks. With the guide, you can compare different tools and analyses, giving health staff the ease of choosing the analysis that best suits your patient. It is important to know that the people without biopsy cannot be diagnosed but they can be treated without the diagnosis to avoid complications. However, we believe that more studies should be carried out in celiac patients to know their relationship with the different pathologies and to be able to expand this guide, making it an aid for health personnel.

Undiagnosed patients with celiac disease symptoms (like diarrhea, heath aches, stomach ache, and anemia) but without the signs (like serological testing and biopsy) should try the gluten-free diet; if his or her health improves, they should keep the gluten-free diet. The reason for this is because we can find several people with silent celiac disease.

## 5. Conclusions

For all the above, it can be said that none of the analyses have a 100% reliability since most of them can present false negatives; therefore, the best way to diagnose CD now is through a combination of different tests such as a study of the patient’s diet, immunoglobulins, and intestinal biopsy. It is expected that, for the future, metabolomics will improve its reliability from 80% to 99% or 100% since it would facilitate the detection of this pathology. To ensure that we do not have false negatives, the patient should eat gluten at least 2 months before the analysis is carried out.

**Figure 2:**
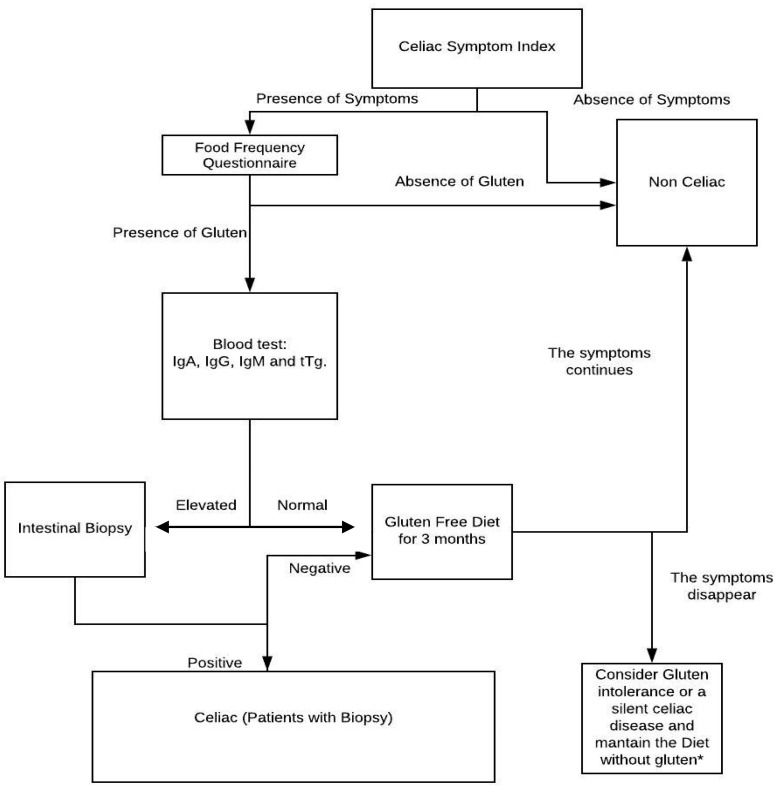
Flow diagram with the suggested steps to diagnose celiac disease. IgA: Immuno-globulin A, IgG: Immuno-globulin G, IgM: Immuno-globulin G, tTg: Transglutaminase anti-inflammatory. * In case the patient has an evident improvement with the treatment (gluten-free diet), it will be continued; otherwise, the presence of other pathologies should be studied.

At the same time, it is recommended that initially noninvasive methods be used for the detection of symptoms as a food reminder or to test if the symptoms vary when stopping the consumption of gluten; in this way, we can discard those patients whose symptoms do not decrease with the treatment. It is recommended that health personnel use a diagnostic scheme like the one indicated in Figure 2 to avoid inflammations and diseases generated by a lack of treatment of CD. With the scheme provided, they can try to avoid false negatives and to improve the quality of life of patients.

In turn, those patients who were already diagnosed with CD and suffer from other symptoms may suffer from any of the related pathologies, such as osteoporosis, cardiovascular diseases, hypothyroidism, etc. Due to this, we created some tables where the health professional can choose the tests that help with the diagnosis of any pathology associated with CD, can try to decrease the search for tests that facilitate the diagnosis, and can make an early treatment that allows the patient to attain a quick recovery. An annual analysis with a food frequency questionnaire and a Celiac Symptom Index questioner are also important, as they always remind the patient the importance of a gluten-free diet.

As a future line of research, it would be very useful to study the new diagnostic tests and to compare them with intestinal biopsy, which is the diagnostic tool for its accuracy. In turn, psychological analyses in this type of patient should be part of more studies, since they show the patient from another very useful perspective so that health personnel can avoid treatment failure.

## Figures and Tables

**Table 1:** Characterization of the articles.

Study	H Index	Age	Amount	Analysis
Churruca et al. [19]	62	16 or +	54	Anthropometric measures, body composition, questionnaires, dietary assessment, serological testing, and intestinal biopsy
Stein et al. [20]	111	20–50	66	Serological testing, tomography, and densitometry
Turner et al. [9]	57	51 ± 16	32	Clinical history, anthropometric measures, densitometry, urine analysis, dietary intake, serological testing, and intestinal biopsy
Roos et al. [21]	13	20 or +	16	Interview and clinical history
Ehsani-Ardakani et al. [22]	18	32 ± 15	130	Clinical history and serum analysis
Fiolkova et al. [23]	14	NA	NA	Clinical history and analysis of literary sources
Rees [24]	45	+18	198	Clinical history and serum analysis
Peterson et al. [25]	27	NA	NA	Physical examination, serological testing, intestinal biopsy, and response to a GFD (Gluten Free Diet)
Belen Zanchetta et al. [26]	61	18–49	53	Serological testing and densitometry
Kocuvan Mijatov et al. [4]	7	23–76	40	Clinical history, questionnaires, and dietary assessment
Santonicola et al. [27]	93	+18	103	Clinical history and questionnaires
Jacobsson et al. [28]	47	20 or +	106	Clinical history and questionnaires
Passananti et al. [10]	42	20–60	95	Clinical history, serum analysis, and anthropometric measures
Fortunato et al. [29]	55	17–49	91	Clinical history
Zanchetta et al. [13]	74	19–50	31	Clinical history, serum analysis, and densitometry
Pinkerton et al. [30]	116	62	1	Clinical history, serum analysis, and densitometry
Jacobsson et al. [2]	47	30–75	54	Interview and clinical history
Singh et al. [31]	102	NA	NA	Clinical history
Tursi et al. [32]	52	22–37	13	Clinical history
Freeman [1]	82	NA	NA	Clinical history and analysis of literary sources
Duerksen et al. [33]	29	20 or +	376	Clinical history, serum analysis, and densitometry
Bykova et al. [34]	11	18 or +	217	Clinical history, serum analysis, and biopsy
Dhalwani et al[7]	142	25–29	2,426,225	Clinical history and analysis of literary sources
Sóñora et al. [35]	37	18–60 (36)	50	Clinical history and serum analysis
Sharshiner et al. [36]	30	30 ± 4.5	232	Clinical history and serum analysis
Ciacci et al. [37]	42	21–45	111	Clinical history, anthropometric measures, serological testing, and genetic analysis
Choi et al. [38]	18	25–39	191	Serological testing with hormones tests and intestinal biopsy
Moleski et al. [39]	24	+18	970	Clinical history, serological testing, intestinal biopsy, and questionnaire
Kavuncu et al. [40]	41	62.75 ± 8.58	192	Clinical history, body mass index, serological testing, biochemical analyses, and densitometry.
Fabbri et al. [41]	129	26	1	Clinical history, brain tomography, serological, biochemical analyses, and cardiovascular analysis.
Ludvigsson et al. [42]	212	Any age	107,124	Clinical history and biopsy
Sonora et al. [43]	37	29–47	10	Clinical history, serological tests, and immunostaining of placental tissue sections
Sultan et al. [44]	234	15–44	276,586	Clinical history
Stephansson et al. [45]	72	+18	66,089	Clinical history and biopsy
Martinelli et al. [46]	40	15–49	248	Clinical history, serum analysis, biopsy, and questionnaire
Tejera-Alhambra et al. [47]	85	29–44	79	Serum analysis
Hasan [48]	–	24–46	50	Clinical history and serum analysis
Mohammadibakhsh et al. [49]	12	NA	NA	Clinical history, serum analysis, biopsy, questionnaire, and analysis of literary sources
Lasa et al. [8]	25	NA	NA	Clinical history, serum analysis, biopsy, questionnaire, and analysis of literary sources
Ludvigsson et al. [50]	114	16 or +	109,327	Clinical history, serum analysis, biopsy, and analysis of literary sources
Ludvigsson et al. [51]	112	NA	173,608	Clinical history, serum analysis, biopsy, and analysis of literary sources

**Table 2:** Diagnostic tests (invasive)/description of the different analyses and tests performed on patients and for what they are used.

Acronym	Name	Instrument for	Objective	Population	Reference Values	Characteristics
-	Intestinal biopsy	Clinical diagnosis of diseases like celiac disease	Observe the tissue	People with an IgA *^1^ elevated and symptomatic (celiac)	Healthy for celiac disease: without the presence of IgA	It is a surgical procedure by which a sample is extracted from tissue to be able to rule out the presence of different pathologies
-	Endoscopy	Observe and obtain tissue samples	Observe and obtain tissue samples	Population at risk or symptomatic	Normal cells	“It is a procedure that allows the doctor to see the inside of your body”. [56]
IgA	Immuno-globulin A	Helps clinical diagnosis of diseases like celiac disease	Measure the amount of immunoglobulin A in blood	Symptomatic people (celiac)	Healthy: 70–350 mg *^5^/dL *^2^ (0.70–3.50 g *^3^/L*^4^)	It is measured in blood serum; it is done through the test ELISA *^5^ and carries few risks. (This description is for IgA, tTg *^6^, IgG *^7^, and IgM *^8^.)
tTg	Transglutaminase anti-inflammatory	Helps clinical diagnosis of diseases like celiac disease	Measure the amount of anti-inflammatory transglutaminase in blood	Symptomatic people (celiac)	They are not specified as they depend on the laboratory and population.	
IgG	Immunoglobulin G	Helps clinical diagnosis of diseases like celiac disease	Measure the amount of immunoglobulin G	Symptomatic people (celiac)	Healthy: 700–1,700 mg/dL (7.0–17.0 g/L)	
IgM	Immunoglobulin M	Helps clinical diagnosis of diseases like celiac disease	Measure the amount of immunoglobulin M	Symptomatic people (celiac)	Will depend on the laboratory that performs the test	
-	Metabolomics	Helps clinical diagnosis of diseases like celiac disease	Measure and study of small molecules	Symptomatic people (celiac)	Presence or absence of certain molecules and proteins for example P-cresol sulfate.	“Is the systematic study of the small molecular metabolites in a cell, tissue, biofluid, or cell culture media that are the tangible result of cellular processes or responses to an environmental stress.” [57]
IRMA *^9^	Immuno-radiometric assay	Clinical diagnosis of diseases like celiac disease	Measure the amount of antigen and labeled antibodies	Symptomatic people	Presence or absence of antigens.	Using florescence, they are labeled with radioactive isotopes of the antibodies in order to find the antigen that is causing the pathology.
-	Genetic Study	Clinical diagnosis of diseases with genetic implication, like celiac disease	Observe the chromosomes	Symptomatic people	Status of chromosomes, absence of mutations.	Uses blood cells to generate cell growth and to be able to study multiple cells during metaphase and, with this, being able to observe the karyotype of each individual and study their DNA *^9^.

*^1^ Immunoglobulin A, *^2^ Deciliters, *^3^ Grams, *^4^ Litter, *^5^ Enzyme-Linked Immuno Sorbent Assay Milligrams, *^6^ Transglutaminase anti-inflammatory, *^7^ Immunoglobulin G, *^8^ Immunoglobulin M, *^9^ Immunoradiometric assay.

**Table 3:** Diagnostic tests (noninvasive)/description of the different analyses and tests performed on patients and for what they are used.

Acronym	Name	Instrument for	Objective	Population	Reference Values	Characteristics
CSI *^1^	Celiac Symptom Index	Monitoring of symptoms	It is made up of sixteen items, 11 of which evaluate “specific symptoms “and 5 evaluate “general health”. [58]	Celiac	More than 45 means	It is a questionnaire-type test.
-	Food Frequency questionnaire	Evaluation of the eating habits	Measure the food groups consumed and its frequency	All the population	WHO values	It is a type of test where the frequency with which the individual consumes certain foods is evaluated.
MDS *^2^	Mediterranean Diet Score	Evaluation of the eating habits compared with the Mediterranean Diet.	Compares the patient’s intake with that recommended in the Mediterranean diet	All the population	The values are between 0 and 55: more points better habits.	It is a questionnaire-type test.

*^1^ Celiac Symptom Index. *^2^ Mediterranean Diet Score.
